# A Brief Review of Current Maturation Methods for Human Induced Pluripotent Stem Cells-Derived Cardiomyocytes

**DOI:** 10.3389/fcell.2020.00178

**Published:** 2020-03-19

**Authors:** Razan Elfadil Ahmed, Tatsuya Anzai, Nawin Chanthra, Hideki Uosaki

**Affiliations:** ^1^Division of Regenerative Medicine, Center for Molecular Medicine, Jichi Medical University, Shimotsuke, Japan; ^2^Department of Pediatrics, Jichi Medical University, Shimotsuke, Japan

**Keywords:** induced pluripotent stem cells, human induced pluripotent stem cells-derived cardiomyocytes, regenerative medicine, 3-dimensional culture, engineered heart tissue

## Abstract

Cardiovascular diseases are the leading cause of death worldwide. Therefore, the discovery of induced pluripotent stem cells (iPSCs) and the subsequent generation of human induced pluripotent stem cell-derived cardiomyocytes (hiPSC-CMs) was a pivotal point in regenerative medicine and cardiovascular research. They constituted an appealing tool for replacing dead and dysfunctional cardiac tissue, screening cardiac drugs and toxins, and studying inherited cardiac diseases. The problem is that these cells remain largely immature, and in order to utilize them, they must reach a functional degree of maturity. To attempt to mimic *in vivo* environment, various methods including prolonging culture time, co-culture and modulations of chemical, electrical, mechanical culture conditions have been tried. In addition to that, changing the topology of the culture made huge progress with the introduction of the 3D culture that closely resembles the *in vivo* cardiac topology and overcomes many of the limitations of the conventionally used 2D models. Nonetheless, 3D culture alone is not enough, and using a combination of these methods is being explored. In this review, we summarize the main differences between immature, fetal-like hiPSC-CMs and adult cardiomyocytes, then glance at the current approaches used to promote hiPSC-CMs maturation. In the second part, we focus on the evolving 3D culture model – it’s structure, the effect on hiPSC-CMs maturation, incorporation with different maturation methods, limitations and future prospects.

## Introduction

The generation of induced pluripotent stem cells (iPSCs) forever changed the field of regenerative medicine, basic and translational biomedical researches (Takahashi et al., 2007). Human iPSCs became an appealing alternative to embryonic stem cells (Bilic and Izpisua Belmonte, 2012; Puri and Nagy, 2012). Since Yamanaka’s breakthrough, many efficient protocols have been developed for generating cardiomyocytes derived from human iPSCs (hiPSC-CMs) (Yang et al., 2008; Elliott et al., 2011; Uosaki et al., 2011; Burridge et al., 2012, 2014; Minami et al., 2012; Dunn and Palecek, 2018).

One of the unresolved problems is that hiPSC-CMs remain largely immature when compared to human adult cardiomyocytes. Such immaturity hinders their usage on many aspects, e.g., pharmacological and toxicological screening (Sinnecker et al., 2014) and cardiovascular disease modeling (Jung and Bernstein, 2014; Yang et al., 2015). Transplanting human embryonic stem cell-derived cardiomyocytes (hESC-CMs) or non-human primate iPSC-CMs into non-human primates model of myocardial ischemia-reperfusion resulted in substantial remuscularization, but non-fatal ventricular arrhythmias were observed (Chong et al., 2014; Shiba et al., 2016), which could be a result of transplanting immature PSC-CMs that have automaticity. To address the issue of immaturity, many different approaches have been examined on enhancing the maturation of hiPSC-CMs.

**FIGURE 1:**
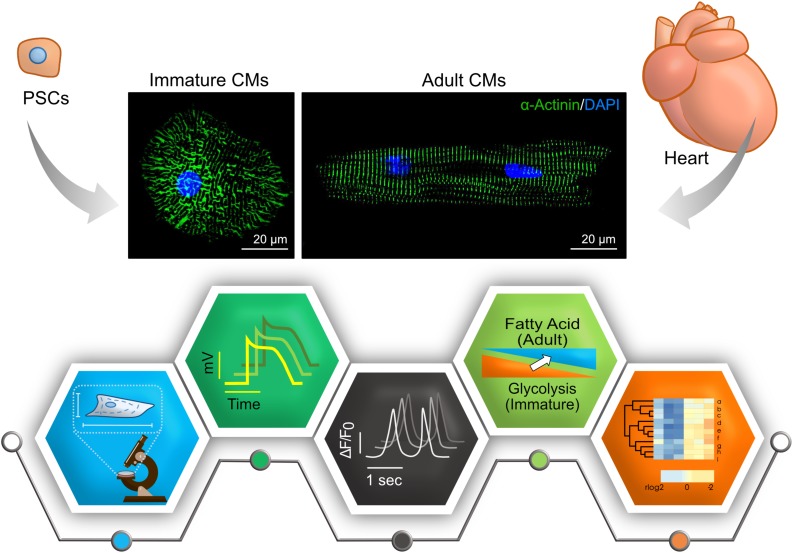
Key features to characterize cardiomyocyte maturity. Compared to adult cardiomyocytes (CMs), many aspects – morphology and structure, electrophysiology, calcium handling, metabolism, and transcriptome – are different in immature PSC-CMs. Representative immunostainings of cardiomyocytes derived PSCs and adult mouse hearts are shown in upper panels (Green, α-actinin; Blue, DAPI). Pictograms shown in lower panels represent each aspect.

**FIGURE 2:**
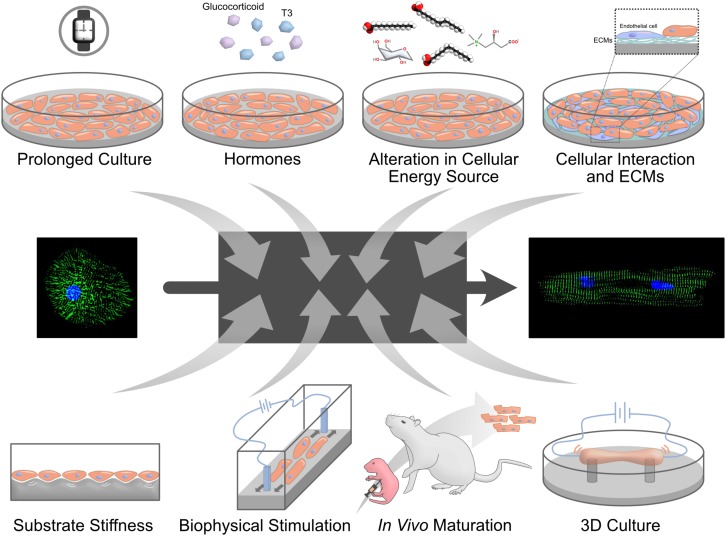
Methods to mature PSC-CMs. Many different approaches to mature PSC-CMs have been proposed. However, it is largely unknown why these signals control cardiomyocyte maturation and how mature PSC-CMs can achieve as a result. A black box shown in the middle represents the unelucidated mechanisms.

In this review, we briefly list the main differences between immature, fetal-like hiPSC-CMs and adult cardiomyocytes (Figure 1). Then, we discuss the current methods used to promote hiPSC-CMs maturation (Figure 2). In the second part, we describe the details of the evolving 3D culture model – it’s structure, the effect on hiPSC-CMs maturation, incorporation with different maturation methods, limitations, and future perspectives.

## Comparison of hiPSC-CMs and Adult Cardiomyocytes to Evaluate Maturity

Researchers in the stem cell field agree that hiPSC-CMs are immature, but there is no consensus about how to evaluate their degree of maturation. Therefore, in addition to developing ways to increase iPSC-CMs maturity, methods on how to assess maturation are required. Here, we summarize characteristics of adult cardiomyocytes and differences to hiPSC-CMs (Figure 1).

### Morphology and Structure

It takes up to 10 years for cardiomyocytes to acquire adult phenotypes in structure and ploidy in a human heart (Peters et al., 1994; Bergmann et al., 2009; Vreeker et al., 2014). Adult cardiomyocytes are well-aligned, rod-like, multinucleated/tetraploid cells, with highly organized sarcomeres, well developed sarcoplasmic reticulum (SR) and transverse tubules (T-tubules) (Peters et al., 1994; Bergmann et al., 2009; Yang et al., 2014a), and have intercalated disks with mature mechanical and electrical junctions (Dhamoon and Jalife, 2005; Zwi et al., 2009; Ma et al., 2011; Kamakura et al., 2013; Vreeker et al., 2014; Denning et al., 2016). Such phenotypical maturation is still lacking in hiPSC-CMs, which tend to be small, mononucleated, rounded cells with disorganized sarcomere. Moreover, they have shorter sarcomeres, poorly developed SR, and no T-tubules (Yang et al., 2014a; Denning et al., 2016). Structural features can be used to evaluate the degree of hiPSC-CMs maturity because some of these features are characteristic to mature cardiomyocytes.

### Physical and Electrophysiological Properties

Adult cardiomyocytes only beat when stimulated with a force around 40–80 mN/mm^2^, conduction velocity around 60 cm/s and upstroke velocity about 150–350 V/s. In hiPSC-CMs, these parameters are around 0.08–4 mN/mm^2^, 10–20 cm/s, and 10–50 V/s, consecutively (Denning et al., 2016). Moreover, hiPSC-CMs display mixed action potential (AP) morphologies that can be categorized as atrial, nodal, or ventricular-like AP (Ma et al., 2011). Although hiPSC-CMs generate important cardiac currents such as I_Na_, I_Ca_,_L_ I_to_, I_Kr_, and I_Ks_, they lack I_K__1_ that is essential for stabilization of the resting potential (Dhamoon and Jalife, 2005; Hoekstra et al., 2012; Knollmann, 2013). This deficiency might be particularly important when hiPSC-CMs are used to study long QT syndrome. Human *ether-a-go-go* related gene (hERG) encode a subunit of I_Kr_ channel, and mutation in hERG or blockade of I_Kr_ cause long QT syndrome. Without I_K__1_, hiPSC-CMs rely on I_Kr_ for the maximum diastolic potential (MDP) that is markedly depolarized with I_Kr_ blockers (Doss et al., 2012). The other characteristic is the spontaneous beatings of hiPSC-CMs. I_f_ current generated by HCN4, which is restricted to pacemaker cells *in vivo*, depolarize MDP and make hiPSC-CMs beat (Yanagi et al., 2007). Measuring the electrophysiological parameters and assessing the generation of I_K__1_ current is a promising tool that can be utilized to assess the maturation of hiPSC-CMs, though it would be technically challenging.

### Calcium Handling

In adult cardiomyocytes, T-tubules and SR are well developed to regulate Ca^2+^ induced Ca release (CICR) and fast excitation-contraction coupling (ECC). The inflow of Ca^2+^ via L-type channels triggers the release of Ca^2+^ from the SR through the ryanodine receptor (RyR) channels (Bers, 2002). T-tubules, invagination of the cell membrane, near L-type Ca^2+^ channel and RyR in adult cardiomyocytes. In the relaxation phase, Ca^2+^ is returned to the SR through sarco/endoplasmic reticulum Ca^2+^ ATPase (SERCA) and is extruded from the cell through the Na^+^–Ca^2+^ exchanger (NCX). The sharp and uniform increase of intracellular Ca^2+^ concentration in adult cardiomyocytes is important for the synchronized contraction in multiple sarcomeres (Scuderi and Butcher, 2017; Steinhoff et al., 2017). In hiPSC-CMs, T-tubules are absent and SR is underdeveloped with low expression of SERCA and other key proteins. As a result, hiPSC-CMs rely on L-type channels for the increase of Ca^2+^ and ECC is slow (Pesl et al., 2017; Veerman et al., 2017).

### Metabolism

In adult cardiomyocytes, mitochondria volume increases, and the oxidative capacity is increased, which represents to switch in metabolic substrates from glucose to fatty acid (Lopaschuk and Jaswal, 2010). To examine glycolysis and fatty acid oxidation, the oxygen consumption rate (OCR) and the extracellular acidification rate (ECAR) are often used, respectively (Rana et al., 2012). During early heart development, around 80% of energy is produced by glycolysis. When cardiomyocytes become mature, fatty acid β-oxidation increases and becomes a major source for energy production. In a rabbit, the metabolic switch occurs during early postnatal growth (Lopaschuk et al., 1991). As hiPSC-CMs remain immature, they rely on glycolysis rather than fatty acid β-oxidation (Rana et al., 2012; Kim et al., 2013; Kikuchi et al., 2015; Correia et al., 2017).

### Gene Expression

Identifying the genes involved in human cardiomyocytes maturation is still an ongoing process. But the overall expression pattern of maturation-related genes identified in mice and humans are mostly similar (DeLaughter et al., 2016; Uosaki and Taguchi, 2016). Isoform transitions of sarcomeric genes occur from fetal to adult period. Cardiac myosin heavy chain (*MHC*, also known as *MYH*) has two isoforms. These are α-isoform (α*-MHC*, also known as *MYH6*) and β-isoform (β*-MHC*, also known as *MYH7*). In adult cardiomyocytes, the β-isoform is predominant. To note, the isoform switch occurs from β-isoform to α-isoform in mouse hearts. Troponin I (TnI) has three isoforms [slow skeletal (ssTnI), fast skeletal (fsTnI), and cardiac (cTnI)] encoded by *TNNI1*, *TNNI2*, and *TNNI3*, respectively. In adult cardiomyocytes, cTnI is highly expressed although ssTnI is the primary isoform in hiPSC-CMs. Titin (*TTN*) has three major isoforms. These are N2B, N2BA and fetal cardiac titin (FCT). In adult cardiomyocytes, N2B is mainly expressed whereas N2BA is predominant in hiPSC-CMs (Yin et al., 2015; Denning et al., 2016). Moreover, hiPSC-CMs show low expression levels of important cardiac genes such as *SERCA2* (sarcoplasmic reticulum ATPase), *CAV3* (caveolin 3), *KCNH2* (potassium voltage-gated channel), and other adult cardiomyocytes genes (Karakikes et al., 2015; van den Berg et al., 2015; Denning et al., 2016).

To assess hiPSC-CMs maturation, measuring the *TNNI3* to *TNNI1* ratio is one way (Bedada et al., 2014). To achieve more precise measurement of maturation, transcriptome-based approaches were proposed, including a gene regulatory network-based (Uosaki et al., 2015) and a relative expression orderings-based scoring method (Chen et al., 2019). Single-cell transcriptome analysis could also predict the developmental ages of cardiomyocytes. However, most of these approaches are limited to mouse PSC-CMs as limited transcriptome data is available for full-spectrum of human developing hearts including late fetal and early postnatal periods (van den Berg et al., 2015; Kreipke et al., 2016; Tiburcy et al., 2017; Cardoso-Moreira et al., 2019). Thus, further work must be done to generate a valid, agreed upon, maturation index for hiPSC-CMs.

## Cues to Promote Maturation of hiPSC-CMs

In order to be able to fully utilize hiPSC-CMs for clinical or research purposes, especially for drug discoveries and disease modeling, they must acquire an adult-like maturation state. In this part, we discuss different approaches used to enhance the maturation of hiPSC-CMs (Figure 2).

### Prolonged Culture Time

It takes years for cardiomyocytes to fully mature *in vivo* (Vreeker et al., 2014), which prompted a hypothesis that prolonged culture time would promote maturation of hiPSC-CMs. To date, hiPSC-CMs were cultured up to a full year to test their maturity (Kamakura et al., 2013; Lundy et al., 2013; Lewandowski et al., 2018). With prolonged culture, hiPSC-CMs displayed more mature phenotypes in morphology (larger cell size), structure (myofibril density, alignment, microscopically visible sarcomere), and physiology (calcium handling and β-adrenergic response). The cells expressed maturation-related cardiac genes such as *MYH7* with isoform switch (Lundy et al., 2013; Lewandowski et al., 2018). Interestingly, extending the cultures to 180 days resulted in more tightly packed myofibrils with the appearance of mature Z-, A-, H-, and I-bands, but not M-bands. M-bands, a key feature of sarcomere structure, are finally developed after 360 days of culture (Kamakura et al., 2013). These results consolidate the fact that prolonged culture generates more mature cardiomyocytes. However, it poses a question if it is possible to yield cells mature enough in a realistic, and financially appropriate culture time frame.

### Biochemical Cues

#### Hormones

Thyroid hormone, known to have a crucial role in cardiac development and cardiovascular physiology (Klein and Ojamaa, 2001), displayed strong enhancement of hiPSC-CM maturation (Yang et al., 2014b). Triiodothyronine (T3) treatment makes hiPSC-CMs bigger, more elongated morphology with longer sarcomeres. T3-treated hiPSC-CMs displayed increased mitochondrial activity and improved calcium handling along with higher contractile force.

Glucocorticoids are essential for maturation of fetal heart structure and function. Endogeneous glucocorticoids work by stimulating glucocorticoid receptor (GR) on fetal cardiomyocytes/vascular smooth muscle to promote myofibril assembly and organization (Rog-Zielinska et al., 2013, 2015). Adding glucocorticoid analog, dexamethasone, to T3 in culture further improve hiPSC-CMs maturation (Parikh et al., 2017).

These results highlight the importance of these chemical cues and call for studying more molecules and combinations that may further enhance maturation of cardiomyocytes.

#### Alterations in Cellular Energy Source

A hallmark of postnatal cardiomyocyte maturation is switching their metabolism from glycolysis to fatty acid oxidation (Yang et al., 2019). Recently, the glucose-free and lactate-containing medium were identified to eliminate non-cardiomyocyte and enrich hiPSC-CMs (Tohyama et al., 2013; Burridge et al., 2014), while hiPSC-CMs are usually cultured in glucose-containing medium. Replacing glucose with galactose and fatty acids – more specifically, palmitate, oleic acid, linoleic acid, and carnitine – enhanced maturation of hiPSC-CMs (Correia et al., 2017; Nakano et al., 2017; Horikoshi et al., 2019; Yang et al., 2019). The switch of energy source not only increased mitochondrial number and metabolisms but also enhanced morphological, structural and physiological maturation. On the other hand, culturing hiPSC-CMs in high glucose medium inhibits their structural and functional maturation by promoting nucleotide biosynthesis (Nakano et al., 2017), which is attributed to a reduction of cardiac glucose uptake and increased nucleotide deprivation during late gestational and early postnatal stages.

### Cellular Interaction and Extracellular Matrices

Cells interact with each other through direct cellular contact or indirect paracrine factors secreted by the neighboring cells in a heart, and cellular interaction has been implicated in cardiac maturation (Talman and Kivelä, 2018; Yoshida et al., 2018; Abecasis et al., 2019). To mimic these cellular interactions *in vitro*, hiPSC-CMs were cocultured with non-cardiomyocytes, such as human mesenchymal stem cells (MSCs) and endothelial cells. Human MSCs secrete VEGF, bFGF, SDF-1, and GM-CSF to mediate differentiation and electrical coupling of hiPSC-CMs (Yoshida et al., 2018). In contrast, endothelial cells express extracellular matrices (ECMs; collagens I and III, fibronectin, thrombospondin-4) to increase sarcomere length of hiPSC-CMs (Abecasis et al., 2019). ECMs alone had some effects on enhancing structural and functional maturation of hiPSC-CMs (Chun et al., 2015; Herron et al., 2016; Ogasawara et al., 2017).

### Substrate Stiffness

Extracellular matrices regulate tissue stiffness, and the stiffness of a heart increases gradually *in vivo* as a result of collagen accumulation (Jacot et al., 2010). This process increases the ability of the heart to pump blood due to its increased stiffness. Compared to hearts (∼10 kPa), cell culture dishes are much stiffer (∼1 MPa), which prompted a hypothesis that soft matrices might be better for the maturation of hiPSC-CMs. Polydimethylsiloxane (PDMS), hydrogels or polyacrylamide (PAA) were often used to create such culture conditions. Soft surfaces (6∼10 kPa) tuned sarcomere tension and contractility, and hiPSC-CMs on it generated more force (0.1 μN) than those on a hard surface (35 kPa, 0.01–2 μN) (Ribeiro et al., 2015). PDMS and PAA are also used to regulate topology of cell morphology and forcing hiPSC-CMs into rectangular shape results in better maturation (Thavandiran et al., 2013; McCain et al., 2014; Ribeiro et al., 2015). In the agreement with this, the vascular structure also forces hiPSC-CMs in a rectangular shape and made them more mature (Vuorenpää et al., 2017).

### Biophysical Stimulation

Biophysical stimulation is absent under a standard culture condition. In a heart, cardiomyocytes are regularly exposed to electrical stimulation and mechanical stress. Applying a continuous electrical stimulation yielded hiPSC-CMs with rod-like morphology, enhanced cellular alignment, and more organized sarcomeres (Chan et al., 2013). Furthermore, subjecting combined synchronized electrical and mechanical stimulation on hiPSC-CM resulted in enhanced localization of N-cadherin toward cell membrane, sarcomere shortening, and reduced transmembrane calcium current, suggesting more mature phenotype (Kroll et al., 2017).

### *In vivo* Maturation

Instead of mimicking *in vivo* environment *in vitro*, an *in vivo* environment can be used to provide all necessary signals including unknown ones to hiPSC-CMs. There are some disagreements what developmental stages of the heart can be appropriate – neonate, adult, or adult heart after infarction (Funakoshi et al., 2016; Cho et al., 2017; Kadota et al., 2017), however, hiPSC-CMs *in vivo* are more matured than ones *in vitro*. Transplantation to neonatal hearts generated adult-like mature hiPSC-CMs within 2 months of transplantation, suggesting that the maturation speed is accelerated in a rat heart and it is defined by non-cell autonomous manner. Furthermore, hiPSC-CMs from a patient of arrhythmogenic right ventricular dysplasia/cardiomyopathy recapitulated disease phenotypes with the neonatal transplantation method (Cho et al., 2017).

## 3D Culture

Cells are aligned three-dimensionally *in vivo* rather than a monolayer. In the last decade, 3D culture methods have been advanced and become an appealing alternative to the conventional 2D monolayer culture for the maturation of hiPSC-CMs. As 3D tissues also resemble native cardiac architecture, 3D tissues of dilated cardiomyopathy hiPSC-CMs could recapitulate disease phenotypes that 2D cultures failed (Hinson et al., 2015). Here, we discuss the usefulness of 3D cultures on the maturation of hiPSC-CMs.

### General Concepts of 3D Culture

Conventional 2D cultures fail to recapitulate the complexity of the *in vivo* cellular crosstalk, tissue architectures, and extracellular microenvironments with forced and unwanted apical-basal polarity. On the other hand, 3D cultures have more similarity to the *in vivo* extracellular microenvironment, support better cellular interactions, and allows for biochemical and physical stimuli to reach the cells in an evenly distributed manner (Duval et al., 2017; Mirbagheri et al., 2019). In 3D cultures, hiPSC-CMs display structural, functional and metabolic maturation compared to that in 2D cultures (Huethorst et al., 2016; Lemoine et al., 2017; Correia et al., 2018; Ulmer et al., 2018). They show improved myofibrillar alignment and sarcolemma remodeling which led to better Ca^2+^ handling (Silbernagel et al., 2020). Moreover, hiPSC-CMs in 3D culture exhibited faster maturation analyzed by transcriptome (Branco et al., 2019), while 2D cultures hinder the maturation or cause maturation arrest (Uosaki et al., 2015).

Different strategies are used to produce 3D cardiac tissues. One way is seeding hiPSC-CMs in to designed scaffolds or embedding them in hydrogel (Lemoine et al., 2017; Correia et al., 2018; Dattola et al., 2019; Silbernagel et al., 2020). To produce scaffold, photolithography is often used, however, it requires clean room and specialized equipment that are not often available for biomedical research labs (Hoang et al., 2018). 3D printing technologies have been evolving rapidly, and digital light processing (DLP)-based printing is now used to fabricate scaffolds or molds to embed cells in hydrogel directly in a cell culture dish (Ma et al., 2019). Another method is layering hiPSC-CMs into multi-layered cardiac tissue constructs, which successfully recapitulate Torsade de Pointes *in vitro* (Kawatou et al., 2017).

Overall, there is a growing effort to learn the appropriate technology and materials to design a reproducible, efficient and affordable 3D culture system, such advancement will be a great step toward the generation of mature, functional cardiomyocytes.

### Enhancing hiPSC-CMs Maturation in 3D Culture Systems

Although 3D cultures of hiPSC-CMs have great potential to make more matured cardiomyocytes, it is insufficient to do so by itself, and a combination of the above-mentioned maturation-promoting methods worked in 2D must be utilized within a 3D setting, which includes cell-cell interaction (e.g., fibroblasts) (Zhang et al., 2017; King et al., 2019; Valls-Margarit et al., 2019; Yan et al., 2019), hormones (Balistreri et al., 2017; Huang et al., 2020), and electrical and physiological stimulation. Below, we will provide a brief review of the electrical and physiological stimulation applied to 3D cultures to reach the ultimate goal.

#### 3D Cultures With Biophysical Stimuli

One of the epoch-making studies was done by Nunes et al. (2013). Embedding hiPSC-CMs with collagen gel into PDMS channel to form a wire-like structure and exposing the tissues to electrical stimuli generate functionally more matured cardiac tissue. To further augment electrical conduction, electrically conductive silicon nanowires or carbon nanotubes were incorporated into hiPSC-CMs spheroids to form an electrically conductive environment (Tan et al., 2015; Roshanbinfar et al., 2019) that is later enhanced by the addition of an exogenous electrical stimulation (Richards et al., 2016). The regimens of electrical stimulation were explored by several groups to achieve T-tubule formation and positive force-frequency relationships (Hirt et al., 2014; Godier-Furnémont et al., 2015). More sophisticated stimulation has been explored, and a high-intensity training regimen of electrical current with gradually increasing frequency, from 2 to 6 Hz within 2 weeks, followed by another week of stimulation at 2 Hz, achieved hiPSC-CMs with adult-like gene expression, well-developed ultrastructure including T-tubule, better calcium handling and contraction force (Ronaldson-Bouchard et al., 2018).

In the mechanical aspect, passive stretch or application of afterload on 3D tissue of hiPSC-CMs (namely, engineered heart muscle or engineered heart tissue) promoted its structural and functional maturation (Abilez et al., 2018; Leonard et al., 2018). A passive stretch is enough to facilitate metabolic switches in hiPSC-CMs (Ulmer et al., 2018). Moderate afterloads are beneficial on cardiomyocyte maturation, while higher afterloads may be detrimental and cause pathological changes (Leonard et al., 2018). The combinations of both electrical and mechanical stimuli – either cyclic stretch or static stress – were also explored (Ruan et al., 2016; LaBarge et al., 2019). In such conditions, hiPSC-CMs displayed a more matured signature than a single stimulus.

## Conclusion and Future Prospects

As we summarized, the maturity of hiPSC-CMs is getting better by numerous efforts. However, methods still need further improvement to reach the desired degree of maturity. Other aspects lacked in this field are to determine definitive maturity of hiPSC-CMs rather than relative measurements and some mechanistic insights why cells are maturing with particular stimuli. Transcriptome-based assay followed by bioinformatics would be one way to define the maturity and uncover mechanistic insights (Uosaki et al., 2015), though more transcriptome data of human hearts in late fetus and postnatal periods is required. As we noted, human cardiomyocytes require years to complete their maturation in a human heart (Vreeker et al., 2014). Therefore, a question remains to be elucidated if and how we can generate adult-like mature hiPSC-CMs at an affordable time and cost. In a specific condition *in vivo*, the maturation process is accelerated (Cho et al., 2017), thus, hopefully, the issue will be resolved in the near future.

## Author Contributions

RA summarized the publications for this mini review and drafted the manuscript. RA, TA, and HU wrote the manuscript. RA and NC drew the figures. HU finalized the manuscript.

## Conflict of Interest

The authors declare that the research was conducted in the absence of any commercial or financial relationships that could be construed as a potential conflict of interest.
